# Factors associated with mosquito control among construction workers: A systematic review

**DOI:** 10.1371/journal.pone.0303330

**Published:** 2024-05-08

**Authors:** Rahmat Dapari, Muhammad Fahmi Mohd Fadzil, Muhammad Yazid Hanzir, Jamal Sham Mohamed Jais, Nur Fatin Safarudin, Adila Albar

**Affiliations:** Department of Community Health, Faculty of Medicine and Health Sciences, Universiti Putra Malaysia Serdang, Selangor, Malaysia; Beni Suef University Faculty of Veterinary Medicine, EGYPT

## Abstract

**Introduction:**

Workers in the construction industry frequently work in construction sites with numerous areas that can potentially accumulate water, such as tanks, wet cement surfaces, or water puddles. These water collection sites become ideal breeding grounds for mosquito infestation, which leads to a higher prevalence of mosquito-borne diseases, especially malaria and dengue among construction workers. Despite that numerous factors have been identified in controlling vector-borne diseases, the specific factors that influence mosquito control at construction sites have yet to be explored.

**Aims:**

This systematic review aims to determine the factors associated with mosquito control among construction workers.

**Methods:**

Primarily, articles related to factors associated with mosquito control among construction workers were collected from two different online databases (ScienceDirect and EBSCOhost). Two independent reviewers were assigned to screen the titles and abstracts of the collected data, stored in Microsoft Excel, against the inclusion and exclusion criteria. Afterwards, the quality of the included articles was critically assessed using the Mixed Method Appraisal Tool (MMAT). Of the 171 articles identified, 4 were included in the final review.

**Results:**

Based on the thorough evaluation, mosquito-related knowledge, practical mosquito prevention measures, and Larval Source Management (LSM) were identified as vital factors associated with mosquito control among construction workers. The significant association between mosquito-related knowledge and control practices indicates higher knowledge linked to effective practices, particularly among female workers and those who were recently infected with malaria. Concurrently, there were notable challenges regarding sustainable preventive measures and larval control methods in construction settings.

**Conclusion:**

Implementing effective mosquito control, including knowledge and practice on mosquito control together with vector control, is highly required to suppress the expanding mosquito population. It is recommended that employers provide continuous mosquito control education and training to their employees and reward them with incentives, while employees should comply with the guidelines set by their employers to ensure successful mosquito control and reduce the spread of mosquito-borne diseases in the construction industry.

## Introduction

Construction workers form an integral segment of a construction team and are frequently tasked to perform physical labour at a construction site. While the term "construction worker" encompasses a wide range of on-field positions, it generally pertains to individuals who carry out various basic construction activities throughout the different stages of a construction project. Some construction workers specialise in specific tasks, including demolishing buildings, removing hazardous materials, constructing highways and roads, excavating tunnels and mine shafts, and installing concrete or asphalt [1]. Concurrently, construction sites often have numerous areas that promote the accumulation of water, such as in tanks, on wet cement surfaces, and in puddles around the construction area. These water collections can become ideal breeding grounds for mosquitoes. Moreover, construction workers are at a higher risk of carrying the malarial parasite, often due to repeated infections associated with substandard living conditions. As a result, construction sites not only provide the necessary breeding environment for mosquito infestation but also a source of the parasites they need. Consequently, urban areas with numerous construction sites exhibit a higher prevalence of mosquito-borne diseases, such as malaria and dengue [2].

Mosquito-borne illnesses continue to pose a major threat to public health worldwide. According to recent statistics, diseases transmitted by vectors contribute to over 17% of all infectious diseases, which result in over 700,000 deaths each year. Malaria contributes a significant portion, with approximately 400,000 deaths annually, many of which are children under 5 years old. Another widespread viral infection spread by Aedes mosquitoes is dengue fever, which puts over 3.9 billion people at risk of infection. Annually, there are an estimated 96 million symptomatic dengue cases globally, leading to around 40,000 fatalities [3]. Numerous factors have been identified to contribute to the effectiveness of mosquito control programs, with socio-economic elements being particularly influential. Certain factors, such as the level of urban development, public awareness, resource accessibility, educational background, and income levels, are crucial in determining the effectual impact of controlling mosquito populations [4]. Environmental factors also significantly influence mosquito breeding and survival, with vital elements, such as water quality, types of vegetation, and urban landscapes, being critical contributors to mosquito proliferation [5]. Besides, climate conditions are another crucial factor affecting mosquito populations. Unpredictable changes in climate and variations in weather patterns greatly influence the growth of these tiny deadly insects. Additionally, the lifecycle and behaviour of mosquitoes rely heavily on other factors, including temperature, humidity, and the amount of rainfall [6].

Knowledge regarding mosquitoes has also been closely related to effective mosquito control practices. Previous studies have shown the crucial role of knowledge, emphasizing the need for widespread awareness and information campaigns that encompass both appropriate practices and efficient mosquito control methods [7]. The aforementioned burden caused by mosquitoes in terms of fatality makes it even more imperative to enforce effective mosquito control and practice. The current mosquito vector control has been outlined by the CDC [8]. For example, in-house mosquito control can be applied via good habits, such as removing standing water and eliminating larvae using larvicide. On a broader scale, community mosquito control can be implemented using aerial spraying of insecticide. Various mosquito control methods can also be integrated and applied, which include using insect repellents, either chemical-based, such as Picaridin, or natural-based, such as oil of lemon eucalyptus. Other personal protective steps that can be taken include using long-sleeved shirts and pants and treating clothes with permethrin [8]. Technically, these mosquito control methods can be applied at construction sites and among construction workers.

According to the recommended guidelines, the primary suggestions for mosquito control practices at construction sites include the general destruction of mosquito breeding sites, using Ultra Low Volume (ULV) spray to disperse chemical control insecticides, and installing environmental modifications, which include using water pumps to avoid standing water and ensuring that the constructed infrastructures have an adequate water drainage system [9].

Despite the widespread presence of vector-borne diseases and the numerous factors involved in controlling these vectors, the specific factors that influence mosquito control at construction sites have yet to be explored. This includes both the characteristics of the workplace location and the workers themselves. Therefore, the objective of this review was to identify the factors associated with mosquito control among construction workers. A systematic review was employed as it is an essential tool to thoroughly understand the diverse factors that contribute to effective mosquito control at construction sites.

## Materials and methods

This systematic review was prepared in accordance with the Preferred Reporting Items for Systematic Reviews and Meta Analyses (PRISMA) updated guideline. The component of mnemonic PEO (population, exposure, outcome) was established as follows:

- Population: construction workers.
- Exposure: factor associated with mosquito control.
- Outcome: mosquito control.

### Searching strategy

The literature search was conducted in the ScienceDirect and EBSCOhost databases from 18 October until 1 December 2023. The following keywords were used when searching for the relevant articles: "construction worker*" OR "construction industr*" AND "mosquito*" AND "control*". All retrieved articles were imported into Google Sheet library, and a library de-duplication was implemented using Microsoft Excel.

### Eligibility criteria

The inclusion criteria for this study were as follows: (1) full publication in the English language; (2) articles were published from 2014 onwards; (3) original articles including cohort, case-control, and cross-sectional investigating the associated factors with mosquito control among construction workers. Meanwhile, the exclusion criteria include the following: mixed method and qualitative studies, as well as non-original articles, such as conference proceedings, perspective, commentary, opinion, reports, systematic reviews, and meta-analyses, were excluded from this study.

### Study selection

Two independent reviewers were in charge of screening the titles and abstracts of the retrieved materials against the inclusion and exclusion criteria. The articles identified during the main screening were kept, and the full-text article was thoroughly reviewed independently by a third reviewer according to the inclusion and exclusion criteria. The fourth reviewer was assigned to resolve any arising disagreements between each of reviewers.

### Critical appraisal and data extraction

The Mixed Method Appraisal Tool (MMAT) was employed to evaluate the quality of articles selected in this study. The MMAT approach includes five core quality criteria for each of the selected articles and focuses on methodological criteria [10]. Basically, an independent reviewer extracts the data, and another independent reviewer then assesses the data. Eligible articles were analysed in detail using the content analysis method without any statistical tests.

## Results

**Fig 1:**
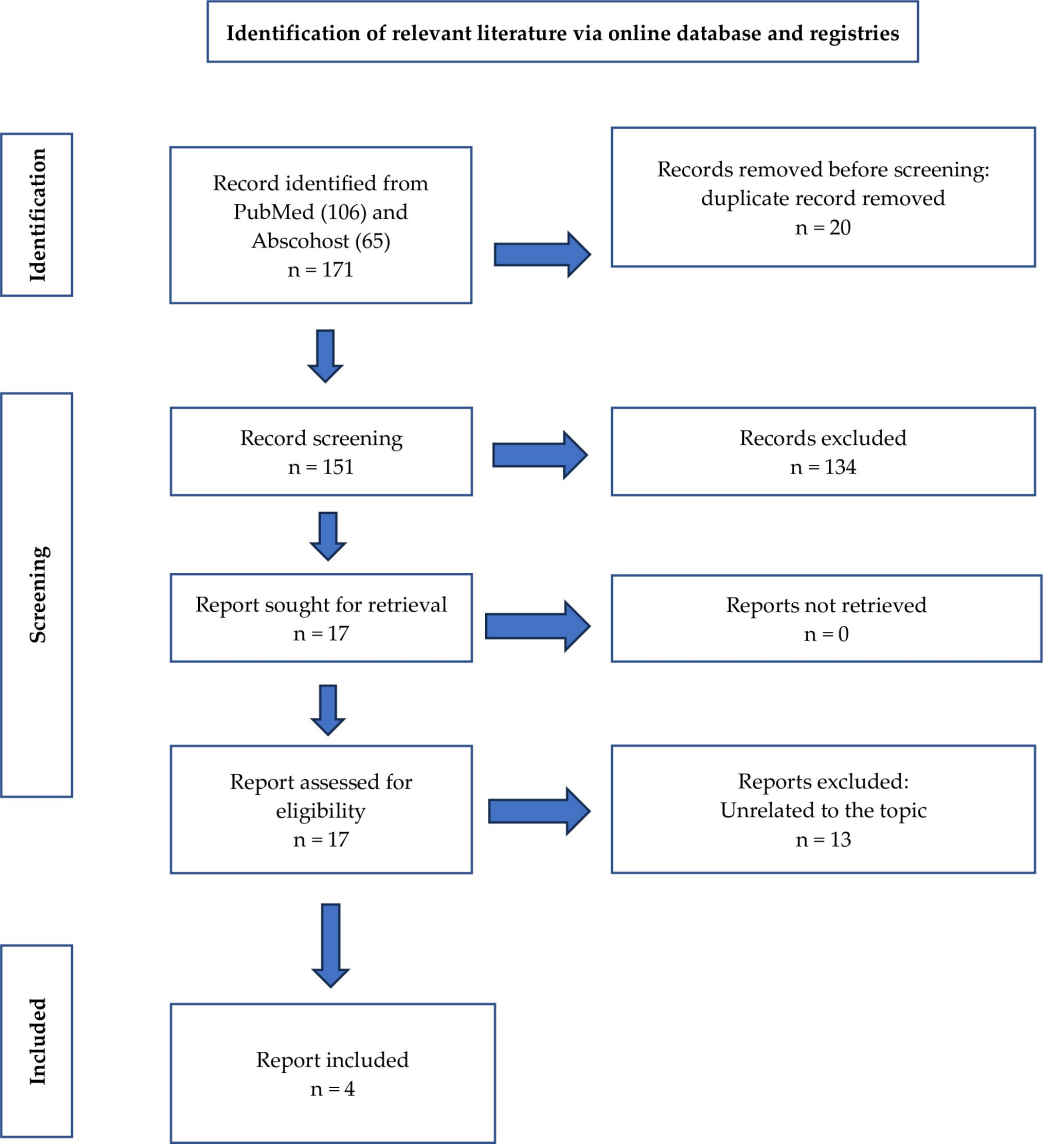
PRISMA flow diagram for the systematic review in this study.

The search strategy process yielded 171 unique hits comprising 106 articles from ScienceDirect and 65 articles from Abscohost as shown in the PRISMA flow diagram (Fig 1). 20 articles were removed during de-duplication process. Subsequently, 151 articles underwent a stringent selection screening process, where each article was meticulously evaluated for its relevance to selection criteria. Following this thorough assessment, only 17 full-text articles were identified as meeting the criteria for further screening process. Following this thorough assessment, 13 articles were removed as they did not meet the inclusion criteria, either due to the study population, exposure or outcome not being related to the focus of this study review. This meticulous approach ensured that only the most pertinent and high-quality literature was included in our review, thereby enhancing the robustness and validity of our findings. A descriptive summary of the included studies in this review regarding the study location and design is presented in Table 1, while Table 2 summarises the findings from the systematic review. The analysed articles were published between 2016 and 2022, whereby three articles were cross-sectional studies, and one article was a case-control study.

**Table 1:** Summary of the study location and study design.

Authors	Study location	Study design
Shivalli et al. (2016) [11]	Mangaluru, India	Cross-sectional
Zou et al. (2021) [12]	West Africa	Cross-sectional
Moore et al. (2017) [13]	Florida, USA	Cross-sectional
Garcia et al. (2022) [14]	Equatorial Guinea	Case-control

**Table 2:** Summary of accepted articles.

Author (Year)	Title	Study design	Sample size	Outcome	Identified factors
Shivalli et al. (2016) [11]	Construction site workers malaria knowledge and treatment-seeking pattern in a highly endemic urban area of India	Cross-sectional	132	Knowledge assessment of malaria among participants	Mosquito-related knowledge: 1. Female workers (β = -0.281, p = 0.001) 2. Self-stated malaria within 1 year (β = 0.276, p < 0.001) 3. Preferred allopathic treatment (β = 0.283, p = 0.001)
Zou et al. (2021) [12]	Study on the use and effectiveness of malaria preventive measures reported by employees of chinese construction companies in Western Africa in 2021	Cross-sectional	256	Malaria infection within 1 year Prevalence: 37.5% of the respondents had been infected with malaria more than once within 1 year	Mosquito preventive measures: 1. Public level: • Free distribution of mosquito nets: 69.9% • Live in a dormitory with a bathroom: 66.0% • Live in a single-person dormitory: 60.1% • Live in a clean and sanitary living environment: 78.1% • Vegetation effectively controlled camp-wide: 22.2% 2. Individual level: • Standardised use of mosquito nets every night: 59.5% • Lighting mosquito coils every day: 49.6% • Wearing long-sleeved shirts and trousers/skirts outdoors: 67.2% • Not saving water indoors: 21.9% • Clean up vegetation around the house every month: 35.9% • Spray insecticide indoors every day: 59.8% Individual practices of mosquito preventive measures were associated with the ordinal measure of infection frequency over the past year with malaria infection: • Standardised use of mosquito nets (p = 0.016) • Spray insecticide (p = 0.047) • Cleaning up the vegetation around self-owned houses (p = 0.028) Public practices of mosquito preventive measures were not statistically associated with malaria infection among employees within 1 year
Moore et al. (2017) [13]	Mosquito control practices and Zika knowledge among outdoor construction workers in Miami-Dade County, Florida	Cross-sectional	49	Mosquito control practice and concerns among construction workers over mosquito-borne diseases	Mosquito preventive measures: 1. Mosquito control practices: • 22% of the survey respondents reported mosquito control activities at their worksite 2. Specific mosquito control and prevention activities: • Personal repellent spray (90.0%) • Standing water treatment, source reduction (clear ditches, tyres), and full-coverage uniform encouragement (20.0%) • Permethrin-treated uniforms or gear (0.0%) 3. Mosquito control activities were done due to • Control nuisance mosquitoes (90.0%) • Preventing mosquito-borne diseases (% not mentioned) 4. A minority of construction employees expressed their concerns about worksite mosquitoes in the previous year (37.5%) 5. The majority of respondents considered mosquitoes a problem to a certain degree at their worksite (76.6%)
Garcia et al. (2022) [14]	The need for larval source management accompanying urban development projects in malaria endemic areas: a case study on Bioko Island	Case-control	4197	Reduced mosquito breeding sites	Larval Source Management (LSM): 1. Treated sites had a significantly lower presence of pupae compared to untreated sites (3.2% vs 18.0%; p < 0.001), with an estimated reduction of 85% (OR: 0.15; 95% COR: 0.12, 0.2) 2. This difference was significant for all habitat types and sizes (p < 0.002), excluding water containers (p = 0.341) BTI-treated sites had a significantly lower presence of late instars compared to untreated sites (14.1 vs 43.6%; p < 0.001), with an estimated reduction of 79% (OR: 0.21; 95% CI OR: 0.18, 0.25), and observed in all habitat types and sizes (p < 0.001)

*Note: β = β value; COR = Crude Odd Ratio; 95% CI = 95% Confidence Interval; p = p-value; BTI = *Bacillus thuringiensis* var. israelensis; LSM = Larval Source Management.

### Common mosquito control

This section describes the outcome of the critical appraisal and data extraction of the four studies, focusing on the standard mosquito control practices among construction workers. The studies included in this review showed significant factors on mosquito control among construction workers, which can be divided into three main factors: (i) mosquito-related knowledge, (ii) Mosquito prevention measures, and (iii) Larval Source Management (LSM).

### Factors associated with mosquito control

#### Mosquito-related knowledge

A community-based cross-sectional study involving 132 respondents was conducted in 9 randomly selected construction sites in the Indian city of Mangaluru, a high-risk urban community for malaria with an annual parasite incidence of > 2/1000/year from June to September 2012 [11]. Female workers had better knowledge scores (β = −0.281, P = 0.001) than male workers, while respondents who had suffered malaria within the past year also displayed better knowledge scores (β = 0.276, p < 0.001). Similarly, those who preferred allopathic treatment had higher knowledge scores (β = 0.283, p = 0.001). In summary, the study identified the factors influencing knowledge scores related to malaria, with female workers, those who had experienced malaria recently, and those preferring allopathic treatment displaying better knowledge.

#### Mosquito prevention measures

Study involving 256 participants, primarily from West African countries, including Nigeria, Mali, Ivory Coast, Ghana, Guinea, Sierra Leone, and Senegal reported that the standardised use of mosquito nets (p = 0.016) and pesticide spraying (p = 0.047) contribute significantly to fewer malaria infections at the individual level [12]. Conversely, the removal of vegetation around residential houses (p = 0.028) at the individual level was associated with a higher incidence of malaria infection [12]. In other words, the use of mosquito nets and pesticide spraying were linked to lower malaria infections, while the removal of vegetation around housing areas was associated with a higher risk of malaria infection at the individual level.

Furthermore, 22% of the survey respondents stated that diverse strategic mosquito control initiatives were implemented at their worksite. In terms of specific mosquito control and prevention measures, personal repellent spray was extensively used, as reported by 90.0% of the respondents. Other practices included treating standing water, engaging in source reduction activities, such as clearing ditches and tyres, and encouraging the use of full-coverage uniforms, accounting for 20.0%. However, none of the respondents (0.0%) utilised permethrin-treated uniforms or gear [13].

The objective behind conducting mosquito control activities was primarily to control nuisance mosquitoes, as cited by 90.0% of the respondents. Preventing the spread of mosquito-borne diseases was also a driving factor, although the percentage was not explicitly mentioned. A minority of the construction employees (37.5%) expressed their concerns over the issue of mosquitoes at the worksite over the past year. Despite the various mosquito control efforts, a majority of the respondents, totalling 76.6%, perceived mosquitoes as a problem to some extent at their worksite [13].

#### Larval source management (LSM)

Study in Bioko Island, Equatorial Guinea identified a total of 4,197 potential larval habitats, of which 3453 potential larval habitats were found at 9 construction sites [14]. The study utilised *Bacillus thuringiensis* var. israelensis (BTI) larvicide to eliminate the larvae at the construction sites, with an average of 384 potential larval habitats per site at each construction site. Additionally, 744 potential larval habitats were identified at two construction sites that were not treated with BTI larvicide. The average number of potential larval habitats per site at these untreated sites was 372. Based on the results, the number of potential larval sites was observed to increase with increasing rainfall at the beginning of the study period. The fluctuation in the number of potential larval habitats ranged between less than 100 and 200 per week, averaging around 150 per week [14].

Additionally, the study reported that the construction sites treated with BTI larvicide had significantly lower observation rates of both pupae (3.2% vs 18.0%; P < 0.001) and late instar *Anopheles* spp. mosquitoes (14.1% vs 43.6%; P < 0.001) compared to untreated sites. *Anopheles* spp. mosquitoes accounted for 67% of mosquitoes collected using human landing collections. Comparatively, a significantly lower number of *Anopheles* spp. mosquitoes were captured in communities adjacent to treated construction sites compared to untreated sites (P < 0.001). The findings also estimated a 38% reduction in the human biting rate in the treated areas (Incidence Rate Ratio (IRR): 0.62, 95% CI IRR: 0.55, 0.69) [14]. Top of Form

### Risk of bias

The quality appraisal of all four quantitative non-randomised studies in this systematic review was conducted using the MMAT based on five main criteria [10]. Details of the MMAT assessment for the selected studies are reported in Table 3.

**Table 3:** The details of the MMAT assessment.

Author	Type of study	1.1	1.2	1.3	1.4	1.5
		Is the sampling strategy relevant to address the research question?	Is the sample representative of the target population?	Are the measurements appropriate?	Is the risk of non-response bias low?	Is the statistical analysis appropriate to answer the research question?
Shivalli et al. (2016) [11]	Quantitative descriptive	Yes	Yes	Yes	No	Yes
Zou et al. (2021) [12]	Quantitative descriptive	Yes	No	Yes	No	Yes
Moore et al. (2017) [13]	Quantitative descriptive	Yes	No	Yes	Yes	No
Garcia et al. (2022) [14]	Quantitative descriptive	Yes	Yes	Yes	No	Yes

## Discussion

This systematic review identified several factors that contributed to the mosquito control practice among construction workers. They were classified into three categories: (i) Mosquito-related knowledge, (ii) Mosquito prevention measures, and (iii) Larval Source Management (LSM).

### Mosquito-related Knowledge

Knowledge of mosquitoes and their disease-carrying capability was found to be significantly associated with mosquito control practices among construction workers [11]. The findings can be related to a study performed in Tibet in 2014, which found that mosquito-related knowledge was significantly associated with mosquito control practice (p < 0.01) [15]. This relationship was also noted in another study in China in 2023 that showed dengue fever knowledge had a direct positive effect on mosquito control behaviour (p < 0.01) [16]. This finding further emphasises the government’s approach to educating and training the community in dengue and its related knowledge, specifically Communication for Behavioural Impact (COMBI). However, the extent of COMBI and its impact needs to be further reviewed since a study in Hulu Langat, Selangor, Malaysia, claimed that COMBI only influenced respondents during its implementation weeks, and the incidence of dengue cases was not reduced [17]. Furthermore, certain construction site approaches can be initiated to address this factor, specifically in the construction industry.

One of the study also showed that female workers (β = -0.281, P = 0.001) were associated with higher malaria knowledge scores [11]. This finding correlates with a recent systematic review that concluded women can be successfully engaged in vector control programmes at the community level [18]. This finding highlighted the need to boost women’s involvement in the construction industry. Since the construction industry is currently dominated by males, reducing this domination may improve mosquito control practices at construction sites [19].

Having experienced malaria within 1 year (β = 0.276, *p* < 0.001) was associated with higher malaria knowledge scores. This finding is in accordance with Kolb’s Experiential Learning Cycle, which states that concrete experience will be reflected and then conceptualised before being experimented to try its success [20]. While it is unethical to expose humans to malaria for experimental purposes, it is more beneficial to provide sufficient training to ensure effective mosquito control practices among construction industry employees.

Furthermore, those preferring modern medicine (β = 0.283, *p* = 0.001) had higher malaria knowledge scores compared to their counterparts. This may be attributed to the relatively advanced health education in India [21]. A similar approach can be used to address the relentless dengue problem in Malaysia.

### Mosquito prevention measures

This review found that individual preventive measures were significantly associated with malaria infection. Specifically, using mosquito nets every night (p = 0.016) and spraying insecticide indoors every day (p = 0.047) was negatively associated with malaria infection. Conversely, cleaning up vegetation around self-owned homes every month had a positive association (p = 0.028) with malaria infection. This may be explained by the potential risk of mosquito bites among those who were involved in the cleaning process, which could lead to malaria infection [12].

Management-initiated practices, which include free distribution of mosquito nets, living in a dormitory with a bathroom, living in a single-person dormitory, living in a clean and sanitary environment, vegetation effectively controlled camp-wide or housing type, were measured in the study in a 1-year duration. However, the results showed that management-initiated practices were insignificantly associated with the infection risk [12]. Hence, this finding warrants further explanation as recall bias may be excluded, given that these activities should have been easy to remember correctly. Although mosquito nets were used every night, the insignificant correlation between the free distribution of mosquito nets and the infection rate indicates that free resources should be used regularly to ensure their effectiveness. In addition, maintaining a good housing area and a clean environment is insufficient to avoid the risk of malaria infection. Instead, self-awareness and worksite behaviour of construction workers are more critical in guaranteeing a safer place to work with minimal risk of malaria infection [12]. Such efforts must be implemented together with individual preventive measures to decrease the incidence of mosquito-borne diseases.

Other factors that require further assessment is the durability of the freely distributed nets. A study in Chad showed that less than one-third of the freely distributed nets were in serviceable conditions after an average of 14 months [22]. Further evidence is needed regarding the effective use of mosquito nets and its association with specific factors that influence the fabric integrity, such as washing frequency, proximity to water for washing, location of kitchen, type of cooking fuel, and low net maintenance [23]. These factors may contribute to the declining effectiveness of the nets, thus explaining the persistent case of mosquito-borne diseases.

### Larval source management (LSM)

LSM refers to the management of aquatic habitats (water bodies) that are potential larval habitats for mosquitoes to prevent the complete growth cycle of immature mosquitoes [24]. For instance, study that employed LSM is significantly reduce the detection of immature mosquitoes in the intervention sites using BTI larvicide compared to control sites [14]. However, health authorities were required to seek approval to enter these construction sites, which makes this approach challenging to be practically applied. Such obstacles can be overcome through legal channels by making it compulsory for construction sites to carry out specific mosquito control measures and facilitating health inspectors in obtaining site visit permits.

Nevertheless, the BTI method has limited effect against Aedes albopictus, which can develop strong resistance to B. thuringiensis, thus rendering it unsustainable [25] Furthermore, B. thuringiensis toxic proteins were relatively weak to kill off the mosquitoes [26].

Alternatively, the construction industry may consider implementing vector control by releasing Wolbachia-carrying mosquitoes at construction sites to control the proliferation of mosquitoes. Through this technique, the Wolbachia-carrying mosquito population will transmit the Wolbachia bacteria to uninfected mosquitoes, causing sterility of mosquitoes, thus reducing their offspring [27]. The release of Wolbachia-carrying mosquitoes at construction sites is expected to be more acceptable since the community responded negatively to the release of Wolbachia-carrying mosquitoes in its initial phase, as previously reported [28].

Another effective alternative is the use of insecticide paint [29]. However, the suitability of this method need to be asssess particularly for a rapidly changing building structure, such as construction sites. Nevertheless, a field trial of insecticide paint in Africa revealed encouraging results in the first three months [30]. However, further study of its effectiveness in the humid and tropical Malaysian environment should be conducted.

### Future recommendations

Successful mosquito control is less achievable using a single approach. Instead, Integrated Vector Management (IVM) combining LSM and practical mosquito prevention measures with other methods is more effective in achieving significant outcomes [31]. As such, employers and employees need to work hand in hand, as recent reports on management-initiated mosquito prevention practices have proven to be unsuccessful if individual measures were not taken concurrently.

Moreover, employers must continue to provide a safe working and living environment for their employees, as the surrounding environment is an integral part of the Epidemiology Triad that affects how a disease spreads. Therefore, preserving the working and living environment should be prioritised, either by the Occupational Safety and Health (OSH) unit or an additional supervisory unit. Continuous employee education should also be in place, as knowledge was significantly associated with mosquito control practice. Besides, employers may consider additional incentives for employees who comply with the proposed preventive measures.

Once the employer has outlined the mosquito prevention measures, employees need to comply with the guidelines. Past evidence showed that employer provision without employees’ involvement would not produce a significant outcome [12]. Hence, individual mosquito prevention measures must be concurrently practised to ensure a higher rate of success. Other novel mosquito control measures, such as the release of Wolbachia-carrying mosquitoes, insecticidal paint, or other methods, should also be considered.

### Review limitations

As with any research, this systematic review is not without limitations. Primarily, the impact of publication bias in this systematic review must be acknowledged, as grey literature was excluded. Furthermore, language bias should also be considered as the search strategy resulted in literature sourced from several countries where English is not the primary language (India, Africa, Equatorial Guinea, and China), but only articles published in English were included. Despite these limitations, this systematic review presented new insights regarding the knowledge, practice, and policy-related factors associated with mosquito control practices among construction workers, which may serve as a valuable guideline for improving service delivery strategies of the dengue control programme in Malaysia.

## Conclusion

The search strategy and MMAT employed in this systematic review revealed three main factors that were associated with mosquito control practices among construction workers: (i) mosquito-related knowledge, (ii) Mosquito prevention measures, and (iii) Larval Source Management (LSM). A significant link between knowledge and effective control practices was observed, especially among female workers and those with malaria experience, which advocates for increased female involvement in the construction industry. While preventive measures, such as the use of mosquito nets, correlated negatively with malaria infections, the concern over their sustainability and efficacy rose over time. Furthermore, LSM methods showed promising outcomes but faced stiff challenges in terms of accessing construction sites and legal action. The findings in this study highlight the complexity of the three factors, which necessitates a unified approach involving both employers and employees to combat mosquito-borne diseases effectively in the construction industry.

## Supporting information

S1 Checklist (DOCX)
